# Contribution of the Nuclear Localization Sequences of Influenza A Nucleoprotein to the Nuclear Import of the Influenza Genome in Infected Cells

**DOI:** 10.3390/v15081641

**Published:** 2023-07-28

**Authors:** Nhan L. T. Nguyen, Wei Wu, Nelly Panté

**Affiliations:** Department of Zoology and Life Sciences Institute, University of British Columbia, Vancouver, BC V6T 1Z3, Canada; juliannguyen@zoology.ubc.ca (N.L.T.N.); wwlyl1985@hotmail.com (W.W.)

**Keywords:** influenza A virus, nucleoprotein, nuclear localization sequence, NLS, nuclear import

## Abstract

Replication of the RNA genome of influenza A virus occurs in the nucleus of infected cells. The influenza nucleoprotein (NP) associated with the viral RNA into ribonucleoprotein complexes (vRNPs) is involved in the nuclear import of the viral genome. NP has two nuclear localization sequences (NLSs), NLS1 and NLS2. Most studies have concentrated on the role of NP’s NLSs using in vitro-assembled or purified vRNPs, which may differ from incoming vRNPs released in the cytoplasm during an infection. Here, we study the contribution of the NP’s NLSs to the nuclear import of vRNPs in a cell culture model system for influenza infection: human lung carcinoma cells infected with viruses containing NP-carrying mutations in NLS1 or NLS2 (NLS2MT), generated by reverse genetics. We found that cells infected with these mutant viruses were defective in the nuclear import of incoming vRNPs and produced reduced amounts of newly synthesized NP, newly assembled vRNP, and progeny virus. In addition, NLS2MT-infected cells were also defective in the nucleolar accumulation of NP, confirming the nucleolar localization role of NLS2. Our findings indicate that both NLS1 and NLS2 have to be present for successful infection and demonstrate the crucial role of these two NLSs in the infection cycle of the influenza A virus.

## 1. Introduction

The influenza A virus is a significant human pathogen causing annual flu epidemics, has caused three major pandemics in the past century, and poses a pandemic threat [1]. This virus belongs to the *Orthomyxoviridae* family, which comprises enveloped viruses with segmented, single-stranded, negative-sense RNA genomes [2]. The influenza A virus genome consists of eight viral RNA (vRNA) segments of variable sizes [3]. In the virion, each RNA segment is associated with multiple copies of the nucleoprotein (NP) and one copy of the trimeric influenza RNA polymerase, forming a rod-shaped viral ribonucleoprotein complex (vRNP) [4]. The influenza RNA polymerase consists of three subunits: polymerase basic protein 1 (PB1), PB2, and polymerase acidic protein (PA) [5].

After viral entry via receptor-mediated endocytosis, the vRNPs are released into the cytoplasm [6,7]. Unlike most RNA viruses, nuclear import is a critical step in the influenza A virus infectious cycle. After they are released in the cytoplasm, incoming vRNPs enter the nucleus of infected cells, and then, newly synthesized NPs enter the nucleus for the initial round of viral genome amplification and the assembly of progeny vRNPs (reviewed by [8]). vRNPs are 50–150 nm in length, depending on the size of the RNA, and their diameters are l5 nm [9]. Since the size limit for passive diffusion through the nuclear pore complex (NPC) is about 5 nm in diameter [10], large molecules such as influenza vRNPs must display at least one nuclear localization sequence (NLS), allowing them to bind to nuclear import receptors of the importin family [11] and be actively imported into the nucleus through the NPC. Although both the NP and the RNA polymerase subunits (PA, PB1, PB2) of the vRNP contain NLSs, the influenza A NP contains two NLSs (NLS1 and NLS2) that direct the nuclear import of both incoming vRNPs and newly synthesized NP [11,12,13]. The latter must enter the nucleus to bind to newly synthesized viral RNAs and assemble progeny vRNPs in the nucleus of the infected cells [14].

NLS1 is a 13-amino acid long unconventional sequence located at the N-terminus of NP (amino acids ^1^MASQGTKRSYEQM^13^) [15] and binds to the importin-α minor NLS-binding site [16]. NLS2 was first proposed to be a classical bipartite NLS [17], but site-directed mutagenesis and crystallographic studies have defined it as monopartite NLS at residues 212–214 (^212^GRKTR^216^) that predominantly binds to the importin-α major NLS-binding site [13]. A recent bioinformatics study found NLS2 to be highly conserved among different influenza A strains, with 67.6% of strains containing ^212^GRKTR^216^ and 31.2% with the sequence ^212^GRRTR^216^ [18]. Performing competition of the nuclear import of NP during infection with chimeric proteins containing NLS1 or NLS2, it was demonstrated that both NLS1 and NLS2 are indispensable for influenza infection [13]. Because both NLS1 and NLS2 are weak micromolar binders of importin-α [13,16] and bind to different importin-α binding pockets, it has been proposed that they can act as a potent import signal if simultaneously bound to importin-α, with NLS1 at the minor and NLS2 at the major NLS-binding pocket of importin-α [13]. Thus, the current model for the nuclear import of influenza NP suggests that NLS1 and NLS2 function in a synergic manner as a bipartite NLS, which forms only in NP’s tertiary (or quaternary) structure [13].

NLS2 is also involved in the nucleolar localization of NP [19,20]. Mutagenesis studies showed that WT NP, but not a mutant NP with alanine substitutions in NLS2, localizes to the nucleolus [19]. Using antibodies against NLS1 or NLS2, it was also demonstrated that NLS2 was exposed both in the nucleus and the nucleolus, while NLS1 was found in the nucleus and not the nucleolus of influenza-infected cells [21]. Hence, NLS2 has a dual role during influenza A virus infection; first, it serves as a functional NLS for the nuclear import of both incoming vRNPs and newly synthesized NP, and then, it is a nucleolar localization signal of NP. However, the nucleolar localization function of NLS is not exclusive to NP, as it has recently been found that the NLS2 sequence is present in the nucleolar protein 14 (NOP14) and plays a role in the nucleolar accumulation of this protein [18].

Most studies have concentrated on the role of NP’s NLSs using in vitro-assembled RNPs or purified vRNPs [11,12,22], which may differ structurally from vRNPs within infected cells. For example, an NLS1 peptide could inhibit the nuclear import of in vitro-generated NP-vRNA complexes in semi-permeabilized cells [12]. Peptide competition and antibody blocking experiments using vRNPs isolated from influenza A virus show that inhibiting either NLS1 or NLS2 reduced the nuclear accumulation of vRNPs in semi-permeabilized cells, which indicates that both NLS1 and NLS2 are needed to mediate effective nuclear import of vRNPs [22].

Similarly, the roles of NLS1 and NLS2 in the nuclear import of NP have been studied using cells transfected with plasmids that express NP or NP with mutations in these NLSs. For example, alanine substitution of basic amino acids of NLS1 impairs the nuclear import NP [12], and a recent study in cells transfected with plasmids expressing NP-carrying mutations in NLS1 or NLS2 shows that NLS1 and NLS2 contribute equally to the nuclear import of NP [18].

Therefore, the contribution of NLS1 and NLS2 to the nuclear import of vRNPs in a naturally-occurring infection system remains to be determined. Fortunately, the influenza virus reverse genetics system has reached a level of sophistication where one can confidently generate recombinant viruses from cloned DNAs, making this system feasible to study the molecular mechanisms of influenza virus replication and pathogenicity (reviewed by [23]). In this system, influenza viral RNAs are transcribed efficiently and amplified by transfecting eight different plasmids into cells in the culture [24]. Compared with studies that evaluate the contribution of NP NLSs in cells expressing NP or with semi-permeabilized cells and in vitro-formed NP-vRNA complexes [12] or purified vRNPs [22], experiments with recombinant viruses allow for the studies of infected cells and eliminate the need for purifying viral proteins for RNP reconstitution in vitro or the isolation of vRNPs.

In this study, we studied the contribution of NLS1 and NLS2 to the nuclear import of the influenza genome and newly synthesized NP in infected cells rather than transfected or permeabilized cells. Experiments with reverse genetic-generated viruses with mutations in NLS1 or NLS2 showed that nuclear import of incoming vRNPs and newly synthesized NP was significantly inhibited in cells infected with the NLS1 and NLS2 mutant viruses than in cells infected with the WT virus. Moreover, NLS2 mutations also affected the nucleolar localization of newly synthesized NP and, therefore, the formation of progeny vRNPs. In summary, our studies with NLS1/NLS2 mutant viruses address the combined roles of the NP’s NLSs during infection.

## 2. Materials and Methods

### 2.1. Cell Culture

Human tumorigenic lung epithelial cells (A549), Madin–Darby canine kidney epithelial (MDCK) cells, and human embryonic kidney (HEK) 293T cells (American Type Culture Collection) were maintained at 37 °C and 5% CO_2_ in Dulbecco’s modified Eagle medium (DMEM) supplemented with 10% fetal bovine serum (FBS), penicillin/streptomycin, 2 mM L-glutamine, and 1 mM sodium pyruvate.

### 2.2. Generation of Influenza A Virus by Reverse Genetics

The eight plasmids contain the cDNA of the influenza A virus strain A/PR8/1934/H1N1 (PHW2000-PA, PHW2000-PB1, PHW2000-PB2, PHW2000-HA, PHW2000-NA, PHW2000-M, PHW2000-NP, and PHW2000-NS) were generously provided by Dr. Honglin Chen (University of Hong Kong) and Dr. Robert Webster (St. Jude Children’s Research Hospital). The Q5^®^ Site-Directed Mutagenesis Kit (New England Biolabs) was used to mutate basic amino acids in NLS1 or NLS2 in the PHW2000-NP plasmid. The two primers used to generate the NLS1 mutant construct were 5′-GATCCAATGGCGTCTCAAGGCACCAAACGATCATATGAACAATGCCG-3′(forward) and 5′-GATCCGGCATTTGTTCATACGATCGTTTGGTGCCTTGAGACGCCATTG-3′(reverse).

The two primers used to generate the NLS2 mutant construct were 5′-GAGGGGTGAAAATGGAGCAAAGACAGCGCCGAATC-3′(forward) and 5′-GATCCGGCGCTGTCTTTGCTCCATTTTCACCCCTC-3′(reverse).

All constructs were confirmed by sequencing. The WT or mutant viruses were generated as previously described [24]. Briefly, a co-culture of MDCK (3 × 10^5^) and HEK-293T (4 × 10^5^) cells were seeded in a 6-well dish and cultured overnight. The next day, the WT NP or mutant NP plasmid and the other seven plasmids were transfected into co-cultured HEK293T/MDCK cells using Lipofectamine 2000 according to the manufacturer’s instructions. The medium was changed to DMEM supplemented with 1% TPCK-trypsin, 2% FBS, penicillin/streptomycin, 2 mM L-glutamine, and 1 mM sodium pyruvate after 24 h post-transfection. Cell culture supernatant containing WT or mutant viruses was obtained 72 h post-transfection and subjected to electron microscopy (EM) or plaque assay.

### 2.3. Electron Microscopy

A 10 µL drop of the supernatant containing the reverse genetic-generated virus was placed on top of a parlodion/carbon-coated copper EM grid that was previously glow-discharged for 30 s. After 8 min, the grid was washed 4 times in drops of distilled water and then negatively stained in 2% uranyl acetate for 1 min. The grid was then visualized using an FEI Tecnai G2 spirit transmission electron microscope (FEI Company, Hillsboro, OR, USA) operated at an accelerated voltage of 120 kilovolts.

### 2.4. Plaque Assay

Plaque assays were used to determine the titer of the virus obtained by reverse genetics and to evaluate progeny virus released from cells after infection. For the latter, the supernatant was obtained from infected A549 cells at 24 and 48 h post-infection. MDCK cells were seeded at high confluency 2–3 days prior to the plaque assay in 6-well plates and deemed appropriate to use in plaque assay when a clear monolayer was established. The infectious supernatants were serially diluted in phosphate-buffered saline (PBS) and added to the monolayers of MDCK cells. Cells were then incubated for 1 h at room temperature in an orbital shaker and shaken at 60 revolutions per minute. Next, the solutions were removed, and cells were rinsed twice with PBS. Afterward, cells were covered with a layer of nutrient agar overlay (1% agarose, 0.1% TPCK-trypsin, and 1% penicillin-streptomycin in minimum Eagle medium). Plates were incubated at 37 °C and 5% CO_2_ for 72 h. Subsequently, the virus was inactivated with Carnoy’s reagent (60% ethanol, 30% chloroform, and 10% glacial acetic acid), and cells were fixed with 4% formaldehyde for 20 min. Cell monolayers were stained with 1% crystal violet (in 20% methanol) for 1 h, rinsed with water, and allowed to air dry to visualize plaques. Non-stained circular spaces were identified as plaques. Plaques were counted and averaged from three separate wells. Viral titers were expressed as a plaque-forming unit (PFU) per ml, which were calculated as follows:

PFU/mL = numbers of plaques per well × dilution/volume of inoculum.

### 2.5. Influenza A Virus Infection

A549 cells were seeded on glass microscope coverslips and then infected with purified reverse genetics-derived influenza A at a multiplicity of infection (MOI) of 5 in DMEM supplemented with 0.2% FBS. Cells were incubated for 15 min at 4 °C to allow the virus to bind to the cell surface. Cells were then moved to 37 °C for 1 h to allow for the virus internalization. After this incubation period, a mild acidic wash (PBS-HCl, pH 5.5 at 4 °C) was performed to exclude the delayed uptake of attached but not internalized virus particles. Subsequently, cells were incubated at 37 °C in DMEM supplemented with 2% FBS according to the times (beyond 1 h) indicated in the figure legends.

#### 2.5.1. Western Blot Analysis

At each desired infection time point, cells were lysed with RIPA buffer (150 mM NaCl, 50 mM Tris-HCl pH 8.0, 0.5 mM EDTA, 0.5% sodium deoxycholate, 0.1% SDS, 0.5% NP-40, 10 mM phenylmethylsulfonyl fluoride, µM pepstatin, 10% µg/mL aprotinin, and 2 mg/mL leupeptin) on ice for 30 min. Lysates were centrifuged at 15,000× *g* for 10 min at 4 °C. The supernatant was diluted with 2× Laemmli sample buffer (62.5 mM Tris-HCl, pH 6.8, 25% glycerol, 2% SDS, 0.01% bromophenol blue, and 5% β-mercaptoethanol) and boiled in a thermomixer at 98 °C for 5 min. An equal amount of protein samples was loaded onto an SDS-PAGE. Proteins were transferred using a Trans-Blot Semi-Dry Electrophoretic Transfer Cell (Bio-Rad) to a polyvinylidene difluoride membrane as described by the instructions provided by the manufacturer. Membranes were then blocked in a blocking buffer (5% skim milk in PBS containing 0.1% Tween 20 (PBST), followed by overnight incubation with different primary antibodies. The protein expressions were detected using primary antibodies against NP (dilution 1:1000, Acris, AM01375PU), M1 (dilution 1:1000, Acris, SM1748P), and β-actin (dilution 1:10,000, Abcam, Ab8227). Next, the membranes were washed three times with PBST and incubated with horseradish peroxidase-conjugated goat anti-mouse IgG secondary antibody (1:10,000; Sigma-Aldrich, A4416) for 1 h at room temperature. After another three washes with PBST, the antibody was detected using Amersham enhanced chemiluminescent Prime Western Blotting Detection Reagent (GE Healthcare, Chicago, IL, USA).

The band intensity was quantified using ImageJ as previously described [25]. Briefly, the rectangle tool drew the same frame around each band. The intensity was then measured by analyzing the grey value inside the frame. In order to compare the protein band of interest, the relative densities were calculated by dividing the band densities of the β-actin loading-control bands.

#### 2.5.2. Immunofluorescence Staining and Imaging of Infected Cells

After each desired infection time point, cells were fixed with 3% paraformaldehyde in PBS for 15 min at room temperature. Cells were then washed with PBS three times, fixed with 3% paraformaldehyde for 15 min, followed by 5 min of permeabilization with 0.2% Triton X-100. Coverslips were incubated in a blocking buffer (PBS containing 2.5% bovine albumin serum (BSA) and 10% goat serum at room temperature for 1 h. After blocking, cells were incubated with an anti-NP antibody (Acris, AM01375PU) diluted at 1:1000 in the blocking buffer for 1 h at room temperature. Next, cells were washed gently three times at 10 min intervals with PBS and then incubated with the goat anti-mouse IgG (H + L) conjugated with Alexa Fluor 568 secondary antibody (Thermo Fisher Scientific, Waltham, MA, USA), and diluted in the blocking buffer for 1 h at room temperature. For some experiments, cells were also incubated with an anti-fibrillarin antibody (Invitrogen, PA5-81171, Waltham, MA, USA) diluted at 1:500 in the blocking buffer for 1 h at room temperature. After blocking, cells were washed gently three times at 10 min intervals with PBS and then incubated with the goat anti-rabbit IgG (H + L) conjugated with Alexa Fluor 568 secondary antibody (Thermo Fisher Scientific), diluted in the blocking buffer, for 1 h at room temperature. Coverslips were then washed three times at 5 min intervals with PBS and mounted with ProLong gold antifade reagent containing DAPI.

All samples were visualized using a Fluoview FV1000 confocal laser-scanning microscope (Olympus Canada Inc., Toronto, ON, Canada) equipped with a 60x/1.42 N.A. (numerical aperture) Olympus UPlanAPO oil immersion objective lens and a photomultiplier detector. To detect DAPI, a 405-diode laser source with an excitation wavelength of 330–385 nm and an emission wavelength of 420 nm was used. To detect Alexa Fluor 568, a green HeNe (helium and neon) laser source with an excitation wavelength of 530–550 nm and an emission wavelength of 575 nm was used.

### 2.6. Quantification of Nuclear Import and Nucleolar Accumulation of NP

Quantification of the nuclear import of NP was performed as described in [22]. Briefly, the mean fluorescent intensity of 20 pixels × 20 pixels areas was measured in the nucleus (Fn) and the cytoplasm (Fc) using ImageJ software version 1.53e (National Institute of Health). The fluorescence of the nuclear envelope was not included in the quantification. After correction for background fluorescence, the results were expressed as the nuclear to cytoplasmic fluorescence (Fn/c) ratio.

Quantification of the nucleolar accumulation of NP was performed similarly and expressed as the ratio of nucleolar to nucleus fluorescence (Fnucleolus/n).

For both quantifications, data were obtained from a total of 85–100 cells per experiment from three independent experiments. Results were analyzed by One-way ANOVA followed by Tukey’s test using GraphPad Prism (GraphPad Software, version 9.1.1). All data are represented as the mean value ± standard error of the mean, and *p* < 0.05 was considered significant.

## 3. Results

### 3.1. Generation of Infectious Influenza A Viruses Carrying NLS1 or NLS2 Mutations in NP

**Figure 1:**
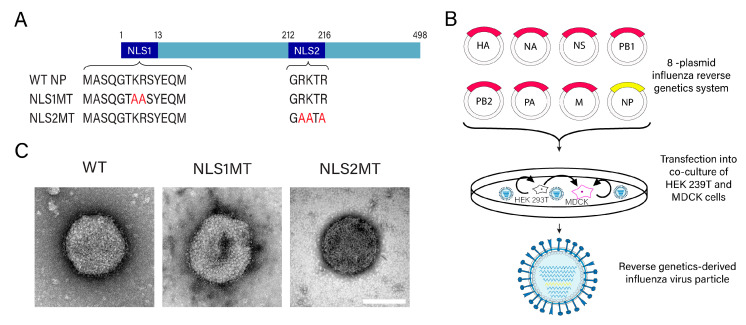
WT and NLS mutant viruses were successfully generated by reverse genetics. (**A**) Schematic representations of the WT NP and its mutants. Alanine substitutions of K or R are shown in red. (**B**) Schematic representations of the influenza A virus reverse genetic system. The eight influenza plasmids are transfected into co-cultured HEK 293T and MDCK cells to generate influenza virus by reverse genetics. For the generation of viruses with mutations in the NLSs of NP, cells were transfected with the reverse influenza genetics system containing HA, NA, NS, PB1, PB2, PA, M, and mutant NP plasmids with alanine substitution, as indicated in panel (**A**). (**C**) Electron micrographs of WT, NLS1MT, and NLS2MT influenza A viruses (generated by reverse genetics) negatively stained with uranyl acetate. Scale bar, 50 nm.

To study the contribution of NLS1 and NLS2 to the nuclear import of vRNPs, recombinant influenza A viruses containing mutations in NLS1 or NLS2 were generated using the eight-plasmid reverse genetic system developed in [26]. These eight plasmids contain full-length cDNAs coding for the viral proteins (HA, NA, PB1, PB2, PA, M, NS, and NP) of influenza A virus strain A/PR/8/34 (H1N1) ligated to the cloning vector pHW2000, as previously characterized [26]. To generate viruses with mutations in the NLSs of NP, site-directed mutagenesis of basic amino acids in NLS1 or NLS2 of the NP plasmid was performed. As illustrated in Figure 1A, two mutants were generated: NLS1 mutant (NLS1MT) containing alanine substitution of K and R at positions 7 and 8 of NLS1 of NP and NLS2 mutant (NLS2MT) containing alanine substitution of K and R at positions 213, 214, and 216 of NLS2 of NP.

WT NP or each of the mutant NP plasmids and the remaining seven plasmids containing cDNAs of influenza A virus (strain A/PR8/1934/H1N1) were transfected into the co-cultured HEK 293T and MDCK cells (Figure 1B). At 72 h post-transfections, supernatants containing WT, NLS1MT, or NLS2MT viruses were collected, and the presence of viruses in the supernatant was determined by EM after negative staining. EM confirmed that WT, NLS1MT, and NLS2MT viruses were successfully generated, and all have similar morphologies (Figure 1C). The viral titers of the genetic-derived influenza A WT and mutant viruses were determined using plaque assays, and the viruses were used for further studies.

### 3.2. Fewer Infectious Viral Particles Are Produced in Cells Infected with NLS1MT or NLS2MT Virus Than in Cells Infected with the WT Virus

**Figure 2:**
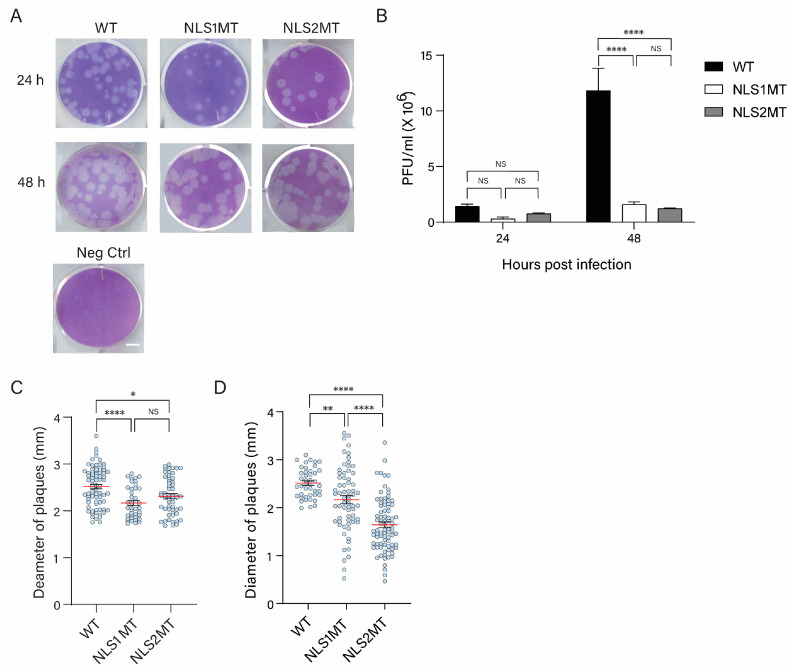
Plaque assays of reverse genetic-derived influenza A viruses at 24 and 48 h P.I. (**A**) Representative example of results of plaque assays. First, A549 cells were infected with reverse genetics-derived WT and mutant influenza A viruses (NLS1MT and NLS2MT). At 24 and 48 h P.I., supernatants from these infected cells were collected and used to perform plaque assay on MDCK monolayers. Scale bar: 5 mm. (**B**) Quantification of the viral titer for cell culture supernatants estimated from three plaque assay replicates performed as described in A. Shown are the means ± standard error of the means scored from three independent experiments. (**C**,**D**) Quantification of the plaque diameter for WT and mutant viruses from plaque assay performed as described in A for cell culture supernatants collected at 24 (**C**) and 48 (**D**) h P.I. Shown are the means (red lines) ± standard error of the means scored from 20-30 plaques for each condition from three independent experiments. (NS, not significant; * *p* < 0.05, ** *p* < 0.01, **** *p*  <  0.0001, one-way ANOVA followed by Tukey’s tests).

To evaluate the infectivity of the reverse genetics-derived WT and mutant viruses, human lung epithelial A549 cells, which represent infected cells found in a physiological influenza A virus infection, were infected with these viruses at an MOI of 5. Then, the supernatants were collected at 24 and 48 h post-infection (P.I.) and subjected to plaque assays. The number of plaques formed was significantly lower for the NLS1MT and NSL2MT viruses than for the WT virus at both 24 and 48 h P.I. (Figure 2B). Moreover, plaques formed by the mutant viruses were significantly smaller than the ones formed by the WT virus from supernatant collected at both 24 and 48 h P.I. (Figure 2C,D). These results suggest that the mutant viruses spread slower than the WT virus to generate distinguishable plaques in the monolayer of MDCK cells. Interestingly, plaques formed by the NLS2MT virus at 48 h P.I. were significantly smaller than those formed by the NLS1MT virus (Figure 2D), indicating that the NLS2MT virus spread slower than the NLS1MT virus.

In summary, these results indicate that fewer infectious viral particles are produced in cells infected with NLS1MT and NLS2MT viruses than in cells infected with the WT virus.

### 3.3. NP and M1 Production Are Delayed in Cells Infected with NLS1MT and NLS2MT Viruses

As plaque assays showed less virus produced in cells infected with NLS1MT or NLS2MT viruses than in cells infected with the WT virus (Figure 2B), we hypothesized that cells infected with mutant viruses might have a defect in the nuclear import of incoming vRNPs. Thus, less vRNA would be transcribed in the host nucleus, and thereby, fewer viral proteins would be produced in cells infected with mutant viruses than in those infected with the WT virus. To test this hypothesis, we performed Western blots to detect newly synthesized NP and M1 inside cells infected with WT or mutant viruses at different times of infection. A549 cells were infected with the WT or mutant viruses. After extensive washing to eliminate virions outside the cells, cell lysates were collected at different times P.I. (2, 5, 8, 10, 12, 24, and 48 h) and subjected to Western blotting using antibodies against NP, M1, and β-actin.

**Figure 3:**
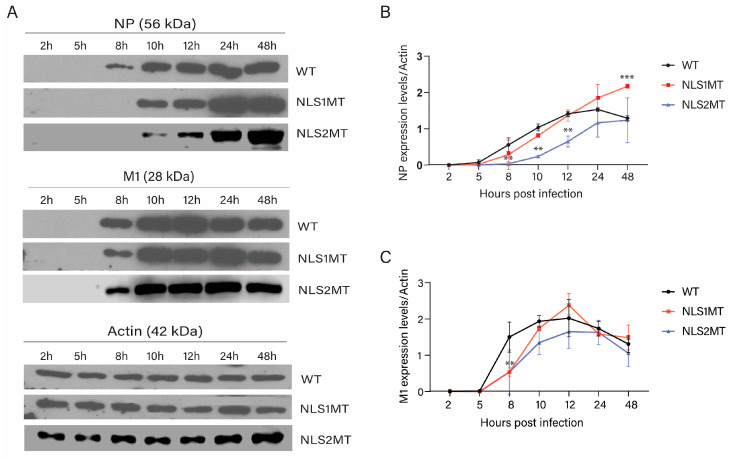
NP production is delayed in NLS1MT- and NLS2MT-infected cells. (**A**) Western blot analysis of NP and actin (loading control) present in the lysate of A549 cells infected with WT, NLS1MT, or NLS2MT viruses at various time points P.I. (**B**,**C**) Quantification of the expression label of NP (**B**) and M1 (**C**) normalized to the amount of β-actin (the band intensity of NP divided by the band intensity of β-actin). Shown are the means ± standard error of the means scored from three independent experiments. (No asterisk denotes non-statistical significance, ** *p*  <  0.01, *** *p* < 0.001, one-way ANOVA followed by Tukey’s tests).

For WT-infected cells, a strong band for NP (56 kDa) started to be detected in the Western blots at 8 h P.I. (Figure 3A). Quantification of the intensity of this band indicated that the expression level of NP increased after 8 P.I. and peaked at 24 h P.I. (Figure 3B). Then, NP production started to decline after 24 h P.I. (Figure 3B). As at this time the infected cells were still intact and we did not detect cell death, this result suggests that progeny viral particles were released from the cells after 24 h P.I.

For cells infected with the NLS1MT virus, an NP band started to be detected at 10 h (Figure 3A), and the intensity of this band continued to rise for all time points tested (Figure 3B), indicating that NP production was delayed in the NLS1MT-infected cells compared to the WT-infected cells. Moreover, significantly less NP was produced in cells infected with the NLS1MT virus than in cells infected with the WT virus at 5, 8, 10, and 12 h P.I. (Figure 3B). However, at 48 h P.I., the amount of NP was significantly higher for cells infected with the NLS1MT virus than those infected with the WT virus (Figure 3B). This result indicates that while cells infected with the WT virus successfully released progeny viruses starting at around 24 h P.I., the NLS1MT-infected cells were delayed in this process, most likely as a consequence of a delay in the replication of the NLS1MT virus.

Similar to NLS1MT-infected cells, an NP band was first observed at 10 h P.I. for NLS2MT-infected cells. Then, the synthesis of NP continued to increase for all time points tested (Figure 3A). However, the production of NP in NLS2MT-infected cells was significantly lower than in cells infected with WT or NLS1MT viruses at 8, 10, and 12 h P.I. (Figure 3B). Thus, the defect in NP production was more severe in NLS2MT-infected cells than in NLS1MT-infected cells.

Production of the matrix protein (M1, 28 kDa), which plays an essential structural and functional role in viral budding from infected cells (reviewed by [27]), had similar defects for cells infected with mutant viruses (Figure 3A,C).

In summary, viral protein production is delayed, and fewer viral proteins are synthesized in cells infected with NLS1MT or NLS2MT viruses than in cells infected with the WT virus. These findings explained the fewer infectious viral particles produced in cells infected with mutant viruses than in cells infected with the WT virus as determined by plaque assays (Figure 2B).

### 3.4. Characterization of Infection Step Defects in Cells Infected with NLS1MT and NLS2MT Viruses

Since our results indicated that NLS1MT- and NLS2MT-infected cells yielded fewer viral proteins (Figure 3) and released fewer progeny virions than WT-infected cells (Figure 2B), the stepwise influenza infection was analyzed in cells infected with WT or mutant viruses using immunofluorescence microscopy to determine the specific steps affected by mutations of NLS1 or NLS2. We hypothesize that the defects in viral protein and progeny virion production are a consequence of a decrease in the nuclear import of incoming vRNPs in cells infected with the NLS1MT or the NLS2MT viruses. We hypothesize that these infected cells will import fewer incoming vRNPs into their nuclei, replicate fewer vRNAs, and produce fewer viral proteins and progeny vRNPs than cells infected with the WT virus. Moreover, we hypothesize that for the NLS2MT-infected cells, the nucleolar localization of newly synthesized NP would also decrease, and fewer progeny vRNPs would be produced. To address these hypotheses, A549 cells were infected with WT, NLS1MT, or NLS2MT influenza A virus at an MOI of 5 for different times (2, 5, 8, 12, 24, and 48 h; to capture different viral infection steps) and prepared for immunofluorescence microscopy using an anti-NP antibody.

NP is a major component of vRNPs; thus, depending on the time and protocol of infection, its immunolabeling during influenza A infection corresponds to the localization of either incoming vRNPs, newly synthesized NP, and/or progeny vRNPs. For example, under our experimental conditions (infection of monolayer A549 cells with purified virions at MOI of 5; see detailed infection protocol in Materials and Methods) at 2 h P.I. with the WT virus, NP immunostaining represents incoming vRNPs, and at 5 h P.I. with the WT virus, NP immunostaining corresponds to newly synthesized NP (because incoming vRNPs enter the nucleus within 10 min of being released from endosomes [28] and are then immediately transcribed to generate the mRNA of NP and synthesize NP). From 8 to 12 h P.I. with the WT virus, NP immunostaining represents both newly synthesized NP and progeny vRNP; thus, we could detect whether progeny vRNPs are localized in the nucleolus and/or have been exported from the nucleus.

To evaluate whether the NP is more concentrated in the nucleus or the cytoplasm, the nuclear to cytoplasmic fluorescence intensity ratio (Fn/c) was quantified as previously described [22,29].

#### 3.4.1. Viral Uptake Is Not Affected, but There Is a Reduction in the Nuclear Import of vRNPs in Cells Infected with the Mutant Viruses

**Figure 4:**
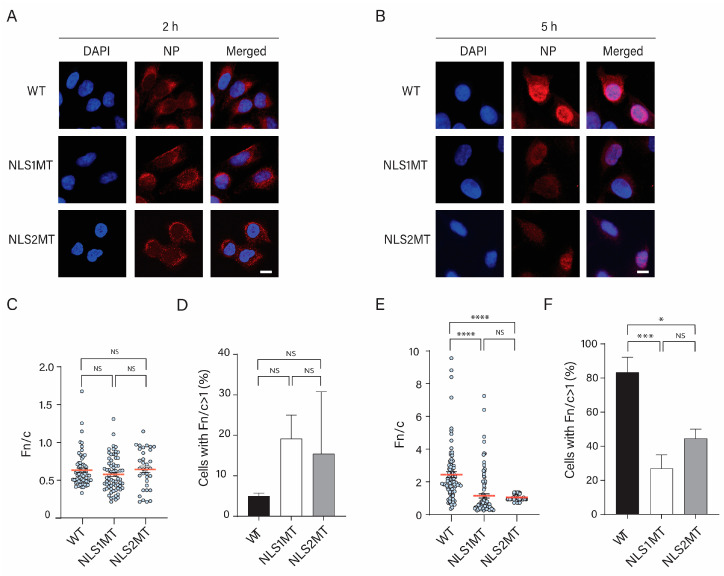
Nuclear import of vRNPs is significantly reduced in cells infected with the mutant viruses. (**A**,**B**) A549 cells were infected with WT or mutant viruses (NLS1MT or NLS2MT) at an MOI of 5 for 2 (**A**) or 5 h (**B**) and immunolabeled with an antibody against NP. Scale bars: 10 µm. DAPI, blue. NP, red. (**C**–**F**) Quantification of the ratio of nuclear to cytoplasmic fluorescence (Fn/c) of NP in WT-, NLS1MT-, and NLS2MT-infected cells at 2 (**C**) or 5 (**E**) h of infection. Quantification of the percentage of cells displaying positive nuclear import of NP (Fn/c > 1) in WT-, NLS1MT-, and NLS2MT-infected cells at 2 (**D**) or 5 (**F**) h of infection. For panels C and E, shown are the means (red lines) ± standard error of the means scored from 85-100 cells for each condition from three independent experiments. For panels D and F, shown are the means ± standard error of the means scored for each condition from three independent experiments. (NS, not significant, * *p* < 0.05, *** *p*  <  0.001, **** *p* < 0.0001, one-way ANOVA followed by Tukey’s tests).

At 2 h P.I., with the WT virus, NP immunolabeling corresponded to internalized incoming vRNPs. Thus, as expected, NP immunostaining of A549 cells infected with the WT virus was detected mainly in the cytoplasm at this time (Figure 4A). The same NP localization was observed for cells infected with mutant viruses (Figure 4A). The quantification of the Fn/c of NP was similar for cells infected with WT and mutant viruses (Figure 4C), indicating that virus uptake was not affected in cells infected with mutant viruses.

For all conditions, only a small percentage of cells have NP in their nuclei (Fn/c > 1; Figure 4D), indicating that nuclear import of incoming vRNPs has started at 2 h P.I. However, there was no significant difference between the percentage of cells with nuclear imported NP for cells infected with WT and mutant viruses at 2 h P.I. (Figure 4D).

At 5 h P.I. with the WT virus, NP immunostaining was detected at higher fluorescence intensity inside the nucleus of infected cells than at 2 h P.I (Figure 4B). This indicates that vRNPs had entered the nucleus and replicated, and NP was produced and entered the nucleus.

For NLS1MT-infected cells, NP immunostaining at 5 h P.I. was also located in the nucleus and was stronger than at 2 h P.I. (Figure 4A,B). Thus, similar to the WT-infected cells, NP was synthesized and entered the nucleus of NLS1MT-infected cells. However, quantification of the Fn/c showed that significantly less NP was in the nucleus of cells infected with the NLS1MT virus than in cells infected with the WT virus (Figure 4E), and there were significantly fewer cells with NP in their nuclei (Fn/c > 1) for NLS1MT-infected cells than for WT-infected cells (Figure 4F).

For NLS2MT-infected cells, the NP fluorescence was lower than for NLS1MT- and WT-infected cells, and both the Fn/c and the number of cells with nuclear NP were significantly lower than for WT-infected cells (Figure 4E,F).

The 5 h P.I. results from WT- and mutant-infected cells suggest that less NP was produced in cells infected with mutant viruses than in cells infected with the WT virus. This could be a consequence of a reduction in the nuclear import of incoming vRNPs in cells infected with the mutant viruses. Moreover, the nuclear import of newly synthesized NP may have also been reduced in NLS1MT- and NLS2MT-infected cells.

#### 3.4.2. Fewer Progeny vRNPs Are Found in the Cytoplasm of Cells Infected with the Mutant Viruses Than in Cells Infected with the WT Virus

**Figure 5:**
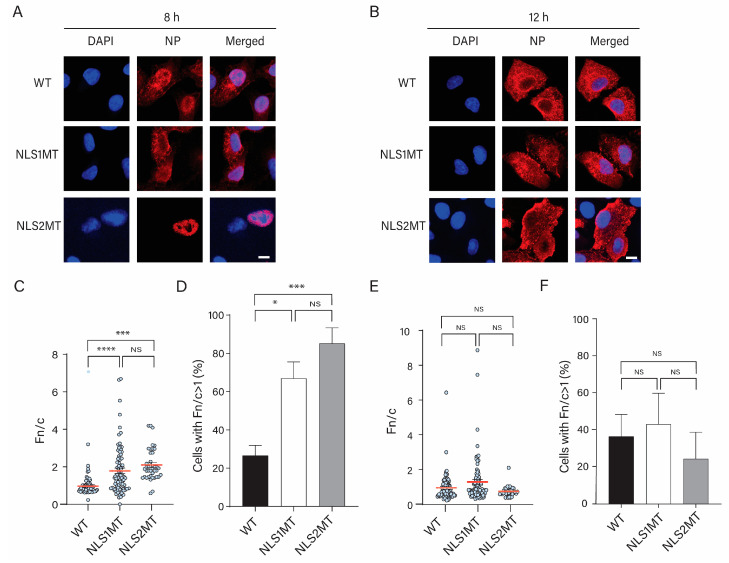
Fewer progeny vRNPs are found in the cytoplasm of cells infected with the mutant viruses than in cells infected with the WT virus. (**A**,**B**) A549 cells were infected with WT or mutant viruses (NLS1MT or NLS2MT) at an MOI of 5 for 8 (**A**) or 12 (**B**) h and immunolabeled with an antibody against NP. Scale bars: 10 µm. DAPI, blue. NP, red. (**C**–**F**) Quantification of the ratio of nuclear to cytoplasmic fluorescence (Fn/c) of NP in WT-, NLS1MT-, and NLS2MT-infected cells at 8 (**C**) or 12 (**E**) h of infection. Quantification of the percentage of cells displaying positive nuclear import of NP (Fn/c > 1) in WT-, NLS1MT-, and NLS2MT-infected cells at 8 (**D**) or 12 (**F**) h of infection. For panels (**C**,**E**), shown are the means ± standard error of the means scored from 85–100 cells for each condition from three independent experiments. For panels (**D**,**F**), shown are the means (red lines) ± standard error of the means scored for each condition from three independent experiments (NS, not significant, * *p* < 0.05, *** *p * <  0.001, **** *p* < 0.0001, one-way ANOVA followed by Tukey’s tests).

At 8 h of infection with the WT virus, NP immunostaining, representing both newly synthesized NP and newly assembled vRNPs, was found in both the nucleus and the cytoplasm (Figure 5A). As for 5 h P.I., nuclear NP corresponds to newly synthesized NP that has entered the nucleus to form progeny vRNPs. At this time, the cytoplasmic NP immunostaining corresponds to progeny vRNPs that have been exported from the nucleus.

For cells infected with the mutant viruses at 8 h P.I., NP immunostaining was generally less strong than those infected with the WT virus (Figure 5A). These results indicate that, in agreement with the Western blot data (Figure 3), less NP was produced in these cells than in cells infected with the WT virus. Quantification of the Fn/c indicated no significant difference between NLS1MT- and NLS2MT-infected cells (Figure 5C). However, there was significantly less NP in the cytoplasm of cells infected with the mutant viruses than in cells infected with the WT virus (Figure 5C). This result indicates that fewer progeny vRNPs are in the cytoplasm of the cells infected with the mutant viruses, most likely because less NP was produced.

Moreover, there were significantly more cells with NP in their nuclei (Fn/c > 1) for cells infected with the mutant viruses than for WT-infected cells (Figure 4D). This result, together with the Fn/c data, indicates a delay in the infection for cells infected with the mutant viruses (while the WT-infected cells have produced enough progeny vRNPs that have been already exported to the cytoplasm at 8 h P.I., most of the cells infected with the mutant viruses have NP in their nuclei, indicating that assembly of progeny vRNPs has not been completed). Consistent with this delay, the Fn/c values and the number of cells with nuclear NP (Fn/c > 1) for cells infected with mutant viruses at 8 h P. I. (Figure 5C,D) are similar to those for WT-infected cells at 5 h P.I. (Figure 4E,F).

At 12 h P.I. with the WT virus, nuclear export of assembled vRNPs was completed as NP immunostaining was detected exclusively in the cytoplasm of WT-infected cells (Figure 5B). In contrast, NP immunostaining was observed in the nucleus and cytoplasm of NLS1MT- and NLS2MT-infected cells (Figure 5B). These results suggest that the nuclear export of vRNPs occurred earlier in WT- than in mutant-infected cells, which is more likely a consequence of a delay in the formation of vRNPs in the nucleus of cells infected with the mutant viruses.

#### 3.4.3. Cells Infected with the Mutant Viruses Have a Delay in Reinfection

**Figure 6:**
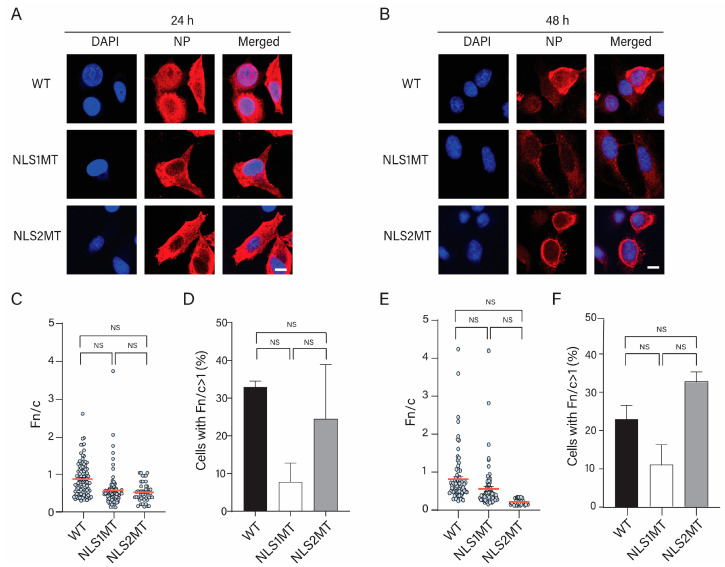
Delay in reinfection of NLS1MT and NLS2MT viruses in A549 cells at 24 and 48 h P.I. (**A**,**B**) A549 cells were infected with WT or mutant viruses (NLS1MT or NLS2MT) at an MOI of 5 for 24 (**A**) or 48 (**B**) h and immunolabeled with an antibody against NP. Scale bars: 10 µm. DAPI, blue. NP, red. (**C**–**F**) Quantification of the ratio of nuclear to cytoplasmic fluorescence (Fn/c) of NP in WT-, NLS1MT-, and NLS2MT-infected cells at 24 (**C**) or 48 (**E**) h of infection. Quantification of the percentage of cells displaying positive nuclear import of NP (Fn/c > 1) in WT-, NLS1MT-, and NLS2MT-infected cells at 24 (**D**) or 48 (**F**) h of infection. For panels (**C**,**E**), shown are the means (red lines) ± standard error of the means scored from 85–100 cells for each condition from three independent experiments. For panels (**D**,**F**), shown are the means ± standard error of the means scored for each condition from three independent experiments (NS, not significant, one-way ANOVA followed by Tukey’s tests).

As at 12 h P.I. with the WT virus, almost all vRNPs have been exported from the nucleus (Figure 5B), NP immunostaining at 24 and 48 h P.I. represents incoming vRNPs of newly generated virions and/or progeny NP/vRNPs of a second or more cycle of infection. Consistent with this, at 24 h P.I., NP immunostaining was detected with high intensity in the nucleus of WT-infected cells (Figure 6A), indicating that several infection cycles were already established at this time point.

For cells infected with the mutant viruses, however, NP immunostaining was detected exclusively in the cytoplasm (Figure 6A), which resembles the NP immunolabeling observed in WT-infected cells at 12 h P.I. (Figure 5B). Because the vRNPs were still in the nucleus of cells infected with mutant viruses at 12 h P.I., the 24 h P.I. results suggest that the nuclear export of progeny vRNPs might be completed in cells infected with the mutant viruses. Thus, contrary to the 24 h P.I. for WT-infected cells, the cells infected with the mutant viruses have not been re-infected.

At 48 h P.I., strong NP immunostaining, which could represent either incoming vRNPs, newly synthesized NP, or/and progeny vRNPs, was found in the cytoplasm of cells infected with the WT virus. (Figure 6B). This result indicates that another round of infection occurred in these cells. In contrast, cells infected with the mutant viruses showed a low cytoplasmic NP fluorescence intensity (Figure 6A), suggesting that reinfection occurred at a low rate in these cells compared to WT-infected cells.

#### 3.4.4. An Overview of the Infectious Cycle of NLS1MT and NLS2MT Viruses

**Figure 7:**
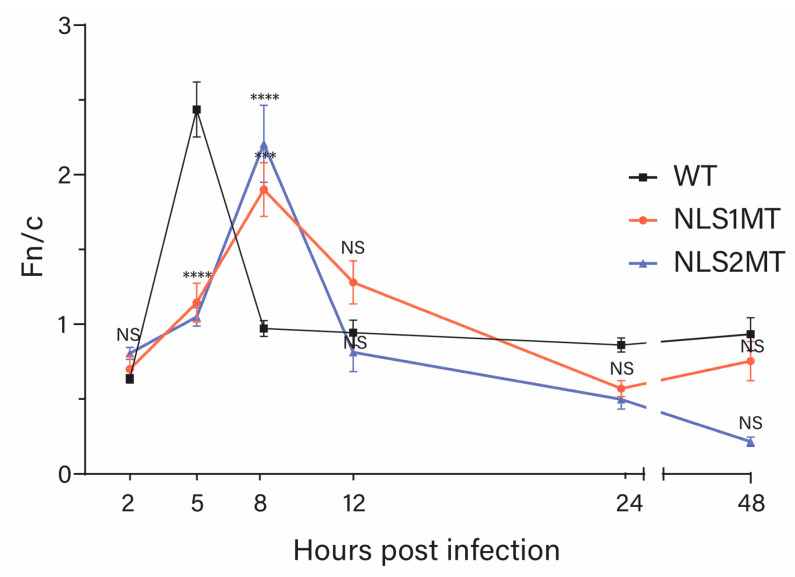
Quantification of the ratio of nuclear to cytoplasmic fluorescence (Fn/c) of NP in WT-, NLS1MT-, and NLS2MT-infected cells at 2, 5, 8, 12, 24, and 48 h P.I. for experiments described in Figure 4, Figure 5 and Figure 6. Shown are the means ± standard error of the means scored from 85–100 cells for each condition from three independent experiments. (NS, not significant; *** *p* < 0.001, **** *p*  <  0.0001, one-way ANOVA followed by Tukey’s tests).

When comparing all the data from all the time points for cells infected with WT and mutant viruses (Figure 7), it was evident that there was a delay in the infectious cycle for the mutant viruses. For example, the Fn/c had a peak at 5 h P.I. for WT-infected cells (Figure 7); this time, it was when the nuclear import of newly synthesized NP reached the highest point. However, this peak was shifted to 8 h P.I. for NLS1MT- and NLS2MT- infected cells (Figure 7). Moreover, at 8 and 12 h P.I., more NP staining was detected in the cytoplasm of cells infected with the WT virus (Fn/c < 1; Figure 7), which suggests that nuclear export of newly assembled vRNP occurred from 8 to 12 h in WT-infected cells. However, Fn/c < 1 occurred only later for cells infected with the mutant viruses (Figure 7).

Although it is more difficult to interpret the comparison of the Fn/c data shown in Figure 7 at later times’ P.I., the NP immunolabeling of cells infected with the mutant viruses at 24 h P.I. (Figure 6A) resembles that of cells infected with the WT virus at 12 h P.I. (Figure 5B). Thus, again the data indicate that mutations in NLS1 or NLS2 delay the infectious cycle of the influenza A virus. This delay is due to reduced nuclear import of both incoming vRNPs and newly synthesized NP in cells infected with the mutant NLS1 and NLS2 viruses. These defects subsequently lead to delays in viral mRNA and protein production, vRNP assembly, nuclear export of newly assembled vRNPs, and release of progeny virions from the infected cells.

### 3.5. Cells Infected with the NLS2MT Virus Have Reduced Nucleolar Localization of NP

Previous studies showed the role of NLS2 in the NP nucleolar localization in cells transfected with plasmids expressing NP or NP with mutations in the NLS2 [19]. In addition, nucleolar localization of NP is essential for the assembly of vRNPs in the nucleolus [20]. Thus, we used the NLS2MT virus to confirm the NLS2 role in the nucleolar localization of NP in influenza-infected-A549 cells rather than in transfected cells.

We hypothesize that mutations of the basic residues of the NLS2 impair the assembly of vRNPs because NLS2 plays a role in the nucleolar localization of NP [19,20]. To address this, the localization of NP and fibrillarin was determined in A549 cells infected with the NLS2MT virus for 8 h, a time at which NP localized to the nucleus of NLS2MT-infected cells (Figure 5A). The localization of these proteins was also detected at 10 h P.I., a time when NP levels are high in NLS2MT-infected cells as detected by Western blots (Figure 3A,B). As a control, the localization of these proteins was also analyzed in A549 infected with the WT and NLS1MT viruses.

**Figure 8:**
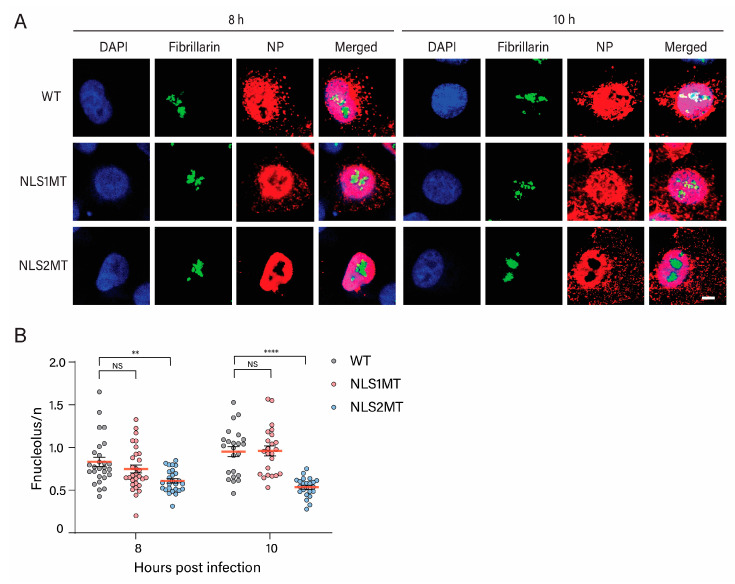
Nucleolar localization of NP in A549 cells at 8 and 10 h P.I. requires a functional NLS2. (**A**) A549 cells were infected with WT or mutant viruses (NLS1MT and NLS2MT) at an MOI of 5 for 8 or 10 h and immunolabeled with antibodies against NP and fibrillarin. Scale bar: 10 µm. DAPI, blue. Fibrillarin, green. NP, red. (**B**) Quantification of the nucleolus to nucleus fluorescence intensity ratio (Fnucleolus/n) of NP at 8 and 10 h P.I. for experiments shown in A. Shown are the means ± standard error of the means scored from 85–100 cells for each condition from three independent experiments. (NS, not significant; ** *p* < 0.01, **** *p*  <  0.0001, one-way ANOVA followed by Tukey’s tests).

Results show a significant reduction in NP in the nucleolus of A549 cells infected with the NLS2MT virus at 8 and 10 h P.I. compared to cells infected with WT and NLS1MT viruses (Figure 8). However, the quantification of the nucleolus to nucleus NP fluorescence intensity ratio (Fnucleolus/n) was similar for both WT- and NLS1MT-infected cells (Figure 8B). These findings demonstrate that NP accumulation in the nucleolus requires a functional NLS2 and show the importance of NLS2 not only for vRNP and NP nuclear import but also in the nucleolar localization of NP.

## 4. Discussion

In this paper, we, for the first time, studied the role of NLS1 and NLS2 in a cell culture model system for influenza infection rather than in transfected cells or permeabilized cells. Following the infection of A549 cells with recombinant influenza A viruses containing mutations in NLS1 or NLS2, we demonstrated that these NLSs must be present for productive infection with the influenza A virus. Cells infected with the mutant viruses had reductions in the production of both viral proteins (Figure 3) and progeny infectious viral particles (Figure 2). Further analyses of infected cells using immunostaining of NP and confocal microscopy demonstrated that these defects in the infection are a consequence of a decrease in the nuclear import of both incoming vRNPs and newly synthesized NP. Notably, the infection defects were more pronounced for the NLS2MT virus than for the NLS1MT virus. We demonstrated that this difference is due to the role of NLS2 in the nucleolar localization of NP, which is needed for the assembly of progeny vRNPs in the nucleolus [20].

Using plaque assays, we found that the mutant viruses exhibited lower infectivity than the WT virus, confirming our hypothesis that mutations in the NLSs of NP affect the nuclear import of both vRNPs and newly synthesized NP. At 48 h P.I., there was no significant difference between cells infected with the NLS1MT and NLS2MT viruses in terms of virus production (Figure 2B), but plaques formed by the NLS2MT virus were significantly smaller than those formed by the NLS1MT virus (Figure 2D). These results indicate that the NLS2MT virus spread slower and was less infectious than the NLS1MT virus. This difference may be due to the role of NLS2 in the nucleolar localization of NP because, in transfected cells, NLS2 significantly promotes the nuclear import of NP to the same extent as NLS1 [18]. Hence, a dysfunctional NLS2 affects the infectious cycle of the influenza A virus more dramatically than a mutated NLS1.

To further explain the low infectivity of mutant viruses observed in plaque assay, we conducted Western blots to detect both NP and M1 in the cell lysates of cells infected with the WT and mutant viruses at different times P.I. Although other proteins, including the viral glycoproteins hemagglutinin (HA) and neuraminidase (NA), could be important in the formation of the virion assembly site, neither of these two viral glycoproteins is critically required for virus assembly, budding, and release because mature virus particles lacking either HA or NA can be formed and released from infected cells [30,31]. In contrast, M1 plays a critical structural and functional role in viral budding from infected cells (reviewed in [27]). Hence, detecting the amount of NP and M1 could allow us to compare the total number of viral proteins not packed in the released virions between WT- and mutant-infected cells. As expected, fewer viral proteins were generated in cells infected with mutant viruses than in cells infected with WT viruses (Figure 3B,C). Based on crystallographic studies, the NLS1 and NLS2 are exposed on the surface of the NP [32] and are not involved in direct interactions with the vRNA [33,34,35], suggesting that the mutations in NLS1 and NLS2 would not destabilize NP or its function in vRNA binding. Hence, the differences in NP and M1 production between cells infected with the WT and mutant viruses observed at early times of infection are due to defects in the early steps of the viral infectious cycle, such as the nuclear import of incoming vRNPs and newly made NP. Because the mutations in the NLS1 or NLS2 may prevent NP from interacting with importin-α to mediate the nuclear entry of incoming vRNPs efficiently, the subsequent steps, such as viral replication and transcription, may also be affected and account for the reduction in viral particles released from infected cells.

Moreover, NP is required for the stabilization and replication of the complementary RNAs (cRNAs) of vRNAs, and NP oligomerization is needed to support the replication [14,36]. Hence, the defects in the nuclear import of newly synthesized NP might further affect viral replication, thus resulting in the overall lower NP and M1 production in cells infected with NSL1MT and NLS2MT viruses than in WT-infected cells. Notably, the production of both NP and M1 remained low in NLS2MT-infected cells compared to WT- and NLS1MT-infected cells (Figure 3). This result indicates that other steps of the NLS2MT virus infectious cycle might be affected, further inhibiting viral replication and infection.

Similar to the Western blot results, the immunofluorescence study showed a lower NP fluorescence intensity at 5 h P.I. in cells infected with mutant viruses compared to those infected with the WT virus (Figure 4). Although there was a significant decrease in the nuclear import of NPs/vRNPs in cells infected with mutant viruses compared to WT-infected cells, some incoming vRNPs from either the NLS2MT or NLS1MT virus still entered the nucleus of infected cells (Figure 4 and Figure 5); thus, cells infected with the mutant viruses could produce progeny virions, but fewer than the WT-infected cells. This is an indication that, although the mutations in NLS1 or NLS2 may affect the nuclear import of incoming vRNPs in NLS1MT-infected or NLS2-infected cells, the remaining functional NLS is still capable of facilitating the translocation of some vRNPs into the nucleus of infected cells. Hence, the replication and transcription of the vRNA still occur to generate new NP but at a much lower extent compared to WT-infected cells because the NLS2 needs NLS1 to work in a synergic manner [13]. This observation indicates that functional NLS1 and NLS2 are both crucial for viral infection, and these NLSs have to work synchronously to obtain the maximum nuclear import of incoming vRNPs and newly made NPs.

We confirmed that NLS2 functions as both an NLS and a nucleolar localization signal of NP, as there was a significant reduction in NP in the nucleolus of A549 cells infected with the NLS2MT virus at 8 and 10 h P.I. compared to cells infected with WT and NLS1MT viruses (Figure 8B). Besides the nuclear import function, other studies have revealed that NLS2 also plays vital roles in viral transcription, replication, and nucleolar accumulation of NP [19,20]. The accumulation of NP in the nucleolus is critical for vRNA replication and vRNP assembly [20], which requires a functional NLS2. Because NP interacts with the RNA polymerase subunits PB1 and PB2, NP may be responsible for accumulating viral polymerase in the nucleolus to facilitate viral replication and progeny vRNP formation [37]. Specifically, multiple basic amino acid changes in NLS2 (R213, K214, and R216) completely impaired vRNA transcription, NP nucleolar localization, and viral replication [19,20]. Mutations in NP’s RNA polymerase binding domain reduce vRNA transcription and replication [38]. These results suggest that NP could serve as the adaptor protein assisting in positioning the viral polymerase complexes to the replication and transcription sites. Hence, when either the NP’s NLS2 or viral polymerase binding site is mutated, the viral polymerase subunits might not be localized to the nucleolus, resulting in incomplete vRNP formation. In support of this hypothesis, our results show that there is less NP and M1 production in cells infected with the NLS2MT virus than in those infected with the NLS1MT virus (Figure 3).

Moreover, in the presence of vRNA, nucleolar fragmentation can be triggered by the co-expression of NP and the viral polymerase subunits to constitute vRNPs [39], and the abundance of a few nucleolar proteins significantly changes during influenza virus infection [40]. For example, the accumulation of influenza A NS1 in the nucleolus during infection causes nucleolin to relocate to the nuclear periphery, and fibrillarin is also redistributed [40,41]. The nucleolar protein RRP1B, associated with the RNA-dependent RNA polymerase and the enhancement of vRNA transcription, is also displaced to the nucleus during influenza virus infection [42]. In addition, the multifunctional proteins of the nucleolus (LYAR) translocate from the nucleolus to the nucleus and cytoplasm, subsequently participating in the assembly of influenza A vRNP complexes [43]. In fact, influenza A is not the only virus that targets nucleolar structure; other DNA and RNA viruses are known to induce nucleolar alterations that contribute to optimal infection [44,45,46]. These findings imply that the nucleolar localization of NP may be crucial for both directing the vRNP complexes to the nucleolus and changing the nucleolar structure to promote viral infection and replication. Therefore, our results, together with previous studies, confirm that NLS2 plays a crucial role in two steps of the influenza A infectious virus cycle (1) to mediate the nuclear import of the incoming vRNP and newly made NP in conjunction with NLS1 and (2) to locate the newly synthesized NP to the nucleolus of infected cells for vRNA replication and the assembly of progeny vRNPs.

## 5. Conclusions

In conclusion, we demonstrated that NLS1 and NLS2 contribute to the nuclear import of incoming vRNP and newly synthesized NPs in infected cells. Furthermore, our results emphasize the importance of NLS2 as a nucleolar localization signal for NP during the influenza virus infectious cycle. Further studies on host factors required for the accumulation of NP in the nucleolus are necessary to understand the detailed mechanisms of vRNA replication and vRNP formation. This knowledge would be a valuable source for better understanding all the steps of the influenza A virus infectious cycle.

## Data Availability

Not applicable.
